# Claudin 18.2, IDH1, and neutrophil-to-lymphocyte ratio as prognostic biomarkers in unresectable pancreatic ductal adenocarcinoma receiving first-line chemotherapy

**DOI:** 10.17305/bb.2026.14093

**Published:** 2026-04-09

**Authors:** Ebru Karcı, Ferhat Ozden, Elif Kuzucular, Ozgur Acikgoz, Ömer Fatih Ölmez, Ozcan Yildiz, Hafize Uzun, Ahmet Bilici

**Affiliations:** 1 Department of Medical Oncology, Faculty of Medicine, Istanbul Atlas University, Istanbul, Türkiye; 2 Department of Medical Oncology, Faculty of Medicine, Istanbul Medipol University, Istanbul, Türkiye; 3 Department of Pathology, Faculty of Medicine, Istanbul Medipol University, Istanbul, Türkiye; 4 Department of Medical Oncology, Faculty of Medicine, Istanbul Acibadem Mehmet Ali Aydinlar University, Istanbul, Türkiye; 5 Department of Medical Oncology, Faculty of Medicine, Biruni University, Istanbul, Türkiye; 6 Department of Medical Biochemistry, Faculty of Medicine, Istanbul Atlas University, Istanbul, Türkiye

**Keywords:** Pancreatic ductal adenocarcinoma, claudin 18.2, isocitrate dehydrogenase, neutrophil-to-lymphocyte ratio, prognosis, overall survival.

## Abstract

Pancreatic ductal adenocarcinoma (PDAC) is a highly aggressive malignancy with few effective treatment options. This study investigates the prognostic significance of claudin 18.2 (CLDN18.2), isocitrate dehydrogenase-1 (IDH1) expression, and the neutrophil-to-lymphocyte ratio (NLR) in patients with unresectable PDAC undergoing first-line chemotherapy. This single-center retrospective analysis utilized prospectively collected data from 72 patients treated with FOLFIRINOX (59.7%) or gemcitabine plus nab-paclitaxel (40.3%). CLDN18.2 and IDH1 expression levels were evaluated through immunohistochemistry, while NLR was calculated from pretreatment blood counts. Survival analyses were conducted using univariable and multivariable Cox regression models. CLDN18.2 positivity was found in 18.1% of patients, and IDH1 positivity was observed in 2.8%. Additionally, an elevated NLR (>3.22) was present in 56.9% of patients. The median progression-free survival (PFS) and overall survival (OS) were 7.1 months (95% CI: 4.2–9.9) and 9.7 months (95% CI: 8.5–10.8), respectively. In the multivariable analysis, CLDN18.2 expression, IDH1 expression, and NLR were significantly associated with OS, whereas no consistent associations were found for PFS. Effect estimates include corresponding 95% confidence intervals. These findings indicate the potential prognostic value of integrating molecular and systemic biomarkers in unresectable PDAC. However, due to the retrospective design and limited sample size, these results should be regarded as hypothesis-generating and necessitate validation in larger, independent cohorts prior to clinical implementation.

## Introduction

Pancreatic ductal adenocarcinoma (PDAC) is the most common form of pancreatic neoplasm and is characterized by its aggressive nature and high mortality rate, largely attributable to challenges in early diagnosis and limited treatment efficacy [1–3]. Despite significant progress in understanding the biology of PDAC and the development of novel therapeutic approaches, the five-year survival rate remains below 10% [4]. Surgical resection is the only potentially curative treatment; however, the majority of patients present with unresectable locally advanced or metastatic disease [5]. Therefore, first-line chemotherapy regimens, such as FOLFIRINOX or gemcitabine combined with nab-paclitaxel, are standard practices aimed at improving survival in patients with unresectable PDAC [4–6]. Immunotherapy is also under investigation, though the inherently non-immunogenic and therapy-resistant microenvironment of PDAC poses significant challenges [7]. Potential immunotherapeutic targets include claudin 18.2 (CLDN18.2), a tight junction protein that is overexpressed in PDAC [8–12]. The therapeutic antibody zolbetuximab, which specifically targets CLDN18.2, is currently undergoing clinical trials [11–13].

Moreover, CLDN18.2 has been identified as a potential prognostic biomarker, as its expression has been significantly correlated with patient survival in previous studies [11, 12]. Another biomarker of interest is the mutant isocitrate dehydrogenase-1 (IDH1 R132H), although its prevalence in PDAC is relatively low [14]. The mutant IDH1 protein may alter cellular redox balance and contribute to resistance under oxidative stress [15]. Pharmacological inhibition of mutant IDH1 is being explored in combination with chemotherapy for various malignancies, including PDAC [15, 16]. Additionally, the neutrophil-to-lymphocyte ratio (NLR), derived from complete blood counts, has been extensively studied as a marker of immune response and clinical outcomes in PDAC [17–19].

Given the poor prognosis associated with unresectable PDAC [1–4], the identification of reliable biomarkers is critical for improving patient stratification and prognostic evaluation. Emerging evidence indicates that IDH1 alterations and CLDN18.2 expression may play significant roles in key oncogenic and metabolic pathways that contribute to tumor aggressiveness. Consequently, this study aims to evaluate the prognostic significance of CLDN18.2 and IDH1 immunohistochemical expression, along with NLR, in patients with unresectable PDAC undergoing first-line chemotherapy.

## Materials and methods

This study was conducted in accordance with the Strengthening the Reporting of Observational Studies in Epidemiology (STROBE) guidelines.

### Patients and procedures

This single-center retrospective study utilized prospectively and consecutively collected data, adhering to the Declaration of Helsinki. The study protocol received approval from the Ethics Committee of Medipol Mega University Hospital, Istanbul, Türkiye (approval number: 10840098-772.02-7244; December 15, 2023), and informed consent was obtained from all participants or their first-degree relatives. Data collection occurred between January 2014 and November 2023.

Patients were consecutively identified from institutional records to minimize selection bias. Inclusion criteria comprised: 1) histologically confirmed unresectable PDAC; 2) receipt of first-line chemotherapy; 3) availability of tumor tissue for immunohistochemical analysis; and 4) baseline complete blood count for NLR calculation. Patients were excluded if they had: 1) a history of other malignancies; 2) pancreatic neuroendocrine tumors; 3) contraindications to chemotherapy; 4) uncontrolled comorbidities; or 5) incomplete follow-up data.

Unresectable disease was defined based on radiological and clinical criteria, including locally advanced tumors with major vascular involvement precluding surgical resection and/or the presence of distant metastases, in accordance with standard clinical practice.

### Chemotherapy protocol

All patients received either the FOLFIRINOX regimen or gemcitabine plus nab-paclitaxel as first-line treatment for a minimum of three months [6]. The FOLFIRINOX protocol was administered biweekly and consisted of 5-fluorouracil (400 mg/m^2^ intravenous bolus on day 1, followed by a continuous infusion of 2400 mg/m^2^ over 46 h), oxaliplatin (85 mg/m^2^ intravenously over 2 h on day 1), and irinotecan (180 mg/m^2^ intravenously over 90 min on day 1). The gemcitabine plus nab-paclitaxel regimen included gemcitabine (1000 mg/m^2^ intravenous infusion over 30–40 min, immediately following nab-paclitaxel on days 1, 8, and 15 of each 28-day cycle) and albumin-bound paclitaxel (nab-paclitaxel; 125 mg/m^2^ intravenous infusion over 30–40 min on days 1, 8, and 15 of each 28-day cycle).

Treatment selection was based on physician discretion, taking into account clinical characteristics such as age, overall health, and comorbidities.

### Data collection

The following variables were collected: age, sex, smoking history, alcohol consumption, weight loss, jaundice at presentation, diabetes status, metastatic status at diagnosis, primary tumor location, visceral metastasis, and chemotherapy regimen. Certain clinical variables, including Eastern Cooperative Oncology Group (ECOG) performance status and detailed comorbidity profiles, were not consistently available and therefore not included in the analysis.

### Immunohistochemistry

Immunohistochemical analysis of CLDN18.2 and IDH1 was conducted on formalin-fixed, paraffin-embedded tumor sections using an automated staining system (Ventana BenchMark ULTRA).

CLDN18.2 expression was evaluated using a semi-quantitative scoring system based on staining intensity (0–3) and the percentage of positive tumor cells. Scoring was primarily based on membranous staining patterns (basolateral and lateral), in accordance with previous studies, while cytoplasmic staining was noted but not used for classification. Samples were considered positive if ≥40% of tumor cells exhibited moderate-to-strong (≥2+) membranous staining [20].

IDH1 staining employed a mutation-specific antibody (clone H09) to detect the IDH1 R132H mutation. Positive staining was defined as granular cytoplasmic reactivity in tumor cells, indicating the presence of mutant protein rather than total IDH1 expression [21].

All slides were independently evaluated by two experienced pathologists.

### Calculation of NLR

NLR was calculated by dividing the absolute neutrophil count by the absolute lymphocyte count prior to chemotherapy [19]. NLR was primarily analyzed as a continuous variable (log-transformed due to skewed distribution). For secondary analyses, patients were dichotomized based on the cohort median (3.22) and a commonly used clinical cutoff (≥3).

### Survival endpoints

Progression-free survival (PFS) was defined as the time from the initiation of chemotherapy to disease progression or death from any cause, whichever occurred first [22]. Overall survival (OS) was defined as the time from treatment initiation to death or last follow-up [22]. Patients were followed at regular intervals post-treatment initiation. Clinical assessments and laboratory tests were performed at each treatment cycle. Radiological evaluations using contrast-enhanced computed tomography were conducted every 8–12 weeks or earlier if clinically indicated. Disease progression was assessed based on radiological and clinical evaluations in routine practice. Progression and death events were recorded during follow-up.

Locally advanced and metastatic cases were analyzed within the same cohort, with metastatic status at diagnosis included as a covariate in multivariable analyses.

**Table 1:** Baseline characteristics of patients with unresectable PDAC (*n* ═ 72)

**Variable**	***n* (%)**
*Age, years*	
≤60	23 (31.9)
>60	49 (68.1)
*Sex*	
Female	33 (45.8)
Male	39 (54.2)
*Smoking history*	
Negative	33 (45.8)
Positive	39 (54.2)
*Alcohol consumption*	
Absent	39 (54.2)
Present	33 (45.8)
*Weight loss at presentation*	
No	28 (38.9)
Yes	44 (61.1)
*Jaundice at presentation*	
No	30 (41.7)
Yes	42 (58.3)
*Diabetes mellitus*	
No	28 (38.9)
Yes	44 (61.1)
*Metastasis at diagnosis*	
No	42 (57.7)
Yes	30 (42.3)
*Primary tumor location*	
Head	33 (45.8)
Body	31 (43.1)
Tail	8 (11.1)
*Visceral metastasis*	
No	47 (64.7)
Yes	25 (35.3)
*First-line chemotherapy*	
FOLFIRINOX	43 (59.7)
Gemcitabine + nab-paclitaxel	29 (40.3)
*CLDN18.2 expression*	
Negative	59 (81.9)
Positive	13 (18.1)
*IDH1 expression*	
Negative	70 (97.2)
Positive	2 (2.8)
*NLR*	
≤3.22	31 (43.1)
>3.22	41 (56.9)

**Abbreviations:** CLDN18.2, claudin 18.2; FOLFIRINOX, folinic acid, fluorouracil, irinotecan, and oxaliplatin; IDH1, isocitrate dehydrogenase-1; nab-paclitaxel, nanoparticle albumin-bound paclitaxel; NLR, neutrophil-to-lymphocyte ratio; PDAC, pancreatic ductal adenocarcinoma.

### Ethical statement

This single-center retrospective study adhered to the principles of the Declaration of Helsinki and utilized prospectively and consecutively collected data. The study protocol received formal approval from the Ethics Committee of Medipol Mega University Hospital (approval number: 10840098-772.02-7244; December 15, 2023). Written informed consent was obtained from all participants or their first-degree relatives prior to study inclusion. All procedures and data collection were performed between January 2014 and November 2023, with patients being consecutively identified from institutional records to minimize selection bias.

**Table 2:** Multivariable Cox regression analysis for progression-free survival (full model output) (*n* ═ 72)

**Variable**	**Category**	**HR (95% CI)**	***P* value**
Age	>60 vs ≤60	1.09 (0.63–1.88)	0.75
Sex	Male vs Female	1.14 (0.67–1.94)	0.63
Smoking history	Positive vs Negative	1.08 (0.62–1.87)	0.79
Diabetes	Yes vs No	1.17 (0.68–2.01)	0.57
Metastasis at diagnosis	Yes vs No	1.31 (0.74–2.30)	0.35
Chemotherapy regimen	Gem+nab vs FOLFIRINOX	1.22 (0.70–2.13)	0.48
CLDN18.2 expression	Positive (reference)	–	–
	Negative	1.48 (0.73–3.01)	0.27
IDH1 expression	Negative (reference)	–	–
	Positive	1.10 (0.05–2.12)	0.98
NLR (continuous, log)	per unit increase	1.32 (0.89–1.96)	0.16

**Abbreviations:** CI, confidence interval; CLDN18.2, claudin 18.2; FOLFIRINOX, folinic acid, fluorouracil, irinotecan, and oxaliplatin; Gem+nab, gemcitabine plus nanoparticle albumin-bound paclitaxel; HR, hazard ratio; IDH1, isocitrate dehydrogenase-1; NLR, neutrophil-to-lymphocyte ratio.

### Statistical analysis

All statistical analyses were conducted using Statistical Package for the Social Sciences (SPSS) version 22 (IBM Corp., Armonk, NY, USA). A two-sided *P* value of <0.05 was deemed statistically significant. Survival outcomes were estimated using the Kaplan–Meier method and compared via the log-rank test. Cox proportional hazards models were employed to assess associations with OS and PFS, reporting hazard ratios (HRs) and 95% confidence intervals (CIs). The NLR was prespecified as a primary variable in survival analyses and modeled primarily as a log-transformed continuous variable to maintain statistical integrity and address its skewed distribution. Sensitivity analyses utilized categorical thresholds based on the cohort median (3.22) and a commonly used clinical cutoff (≥3) to enhance clinical interpretability. Variables were selected based on both statistical criteria (*p* < 0.20 in univariable analysis) and clinical relevance, with multivariable models adjusted for metastatic status and chemotherapy regimen. Due to the limited sample size and low number of IDH1-positive cases, analyses were exploratory in nature. The proportional hazards assumption was evaluated using graphical methods and Schoenfeld residuals, with no adjustments made for multiple comparisons.

## Results

### Patient characteristics

A total of 72 patients with unresectable PDAC were included in the analysis. Baseline demographic and clinical characteristics are summarized in Table 1. The mean age of participants was 63.5 years, with 54.2% being male. At diagnosis, 42.3% of patients presented with metastatic disease. CLDN18.2 positivity was observed in 18.1% of patients, while IDH1 positivity was rare (2.8%). An elevated NLR (>3.22) was found in 56.9% of the cohort. Effect estimates are presented alongside corresponding 95% confidence intervals to indicate the degree of uncertainty.

**Figure 1.**
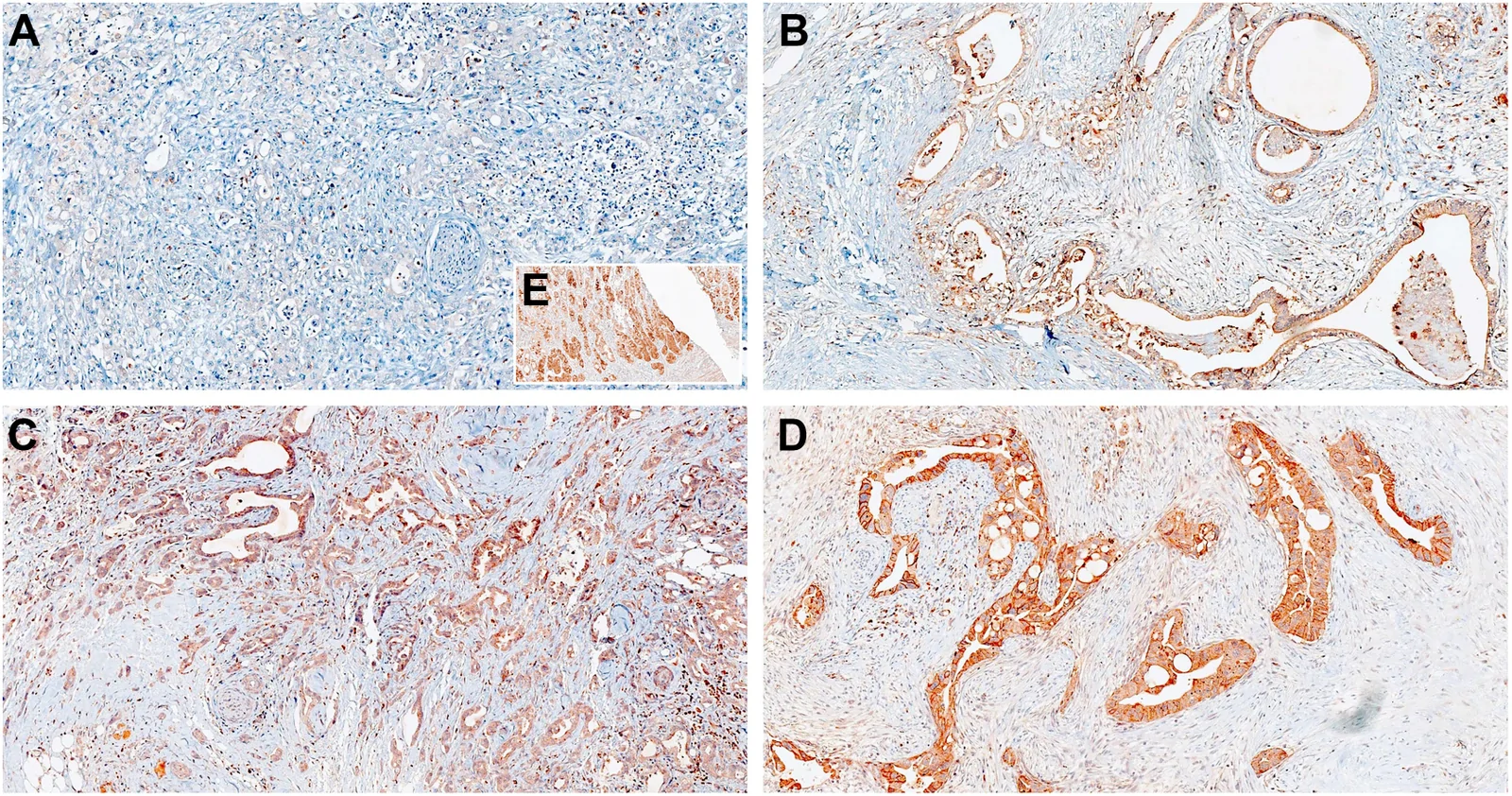
**Immunohistochemical expression of CLDN18.2 in pancreatic ductal adenocarcinoma**. Representative images illustrating the intensity component of the semi-quantitative CLDN18.2 scoring system used in this study: (A) score 0 (negative), (B) score 1+ (weak), (C) score 2+ (moderate), and (D) score 3+ (strong). Scoring was based primarily on basolateral and lateral membranous staining in tumor cells, whereas cytoplasmic staining, when present, was recorded but not used for classification. In the study cohort, tumors were considered CLDN18.2-positive when ≥40% of tumor cells exhibited moderate-to-strong (2+ or 3+) membranous staining. Inset (E) shows normal gastric mucosa used as the positive control. **Abbreviations:** CLDN18.2, claudin 18.2; PDAC, pancreatic ductal adenocarcinoma.

**Figure 2.**
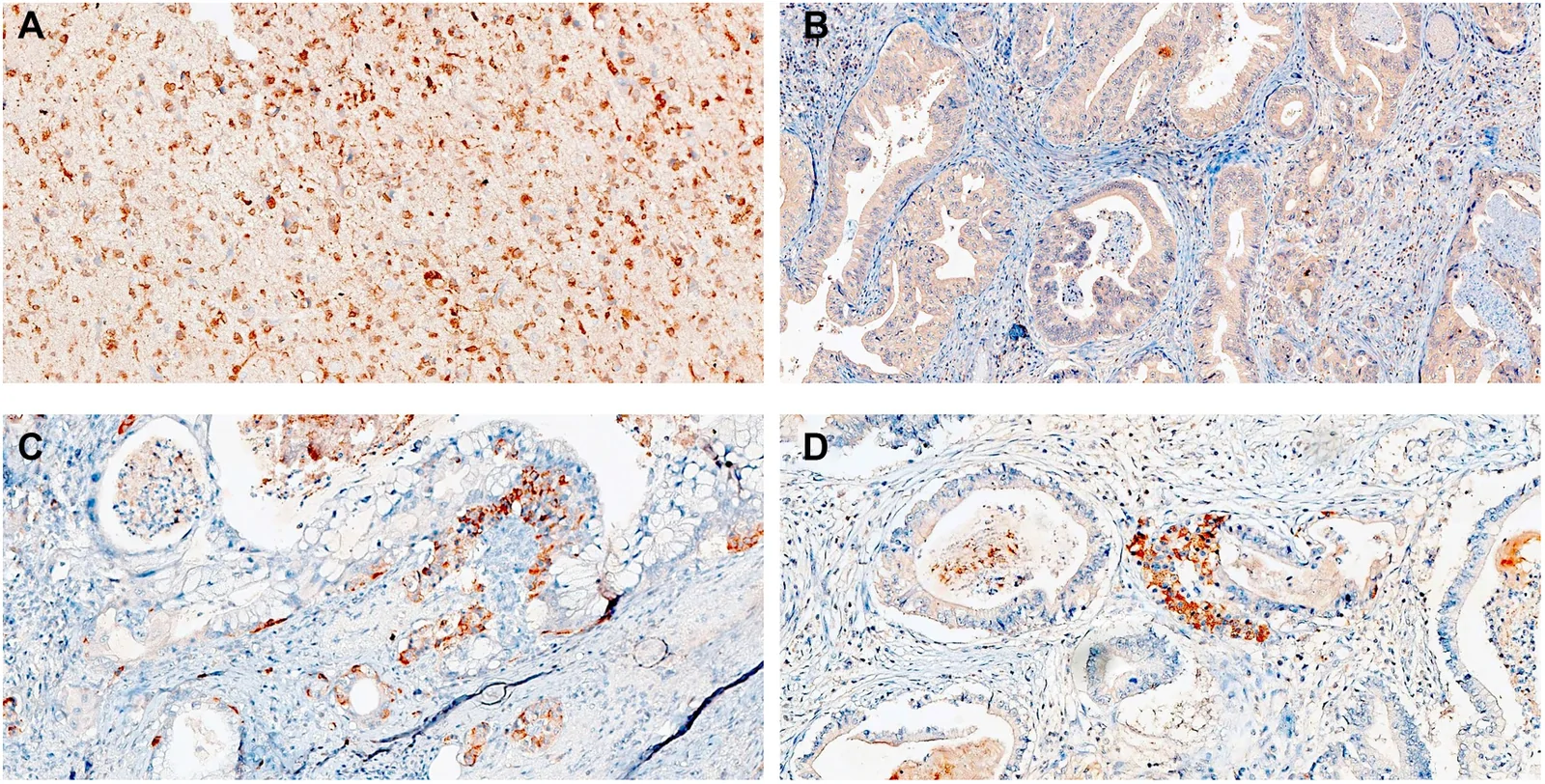
**Immunohistochemical detection of mutant IDH1 R132H in pancreatic ductal adenocarcinoma**. Representative immunohistochemical staining performed with the mutation-specific anti-IDH1 R132H antibody (clone H09). (A) Positive control (oligodendroglioma) showing diffuse granular cytoplasmic staining. (B) Negative PDAC sample without convincing granular cytoplasmic reactivity in tumor cells. (C–D) Representative positive PDAC cases demonstrating focal granular cytoplasmic staining in tumor cells, which was interpreted as evidence of IDH1 R132H mutant protein rather than total IDH1 expression. **Abbreviations:** IDH1, isocitrate dehydrogenase 1; PDAC, pancreatic ductal adenocarcinoma.

### Progression-free survival

The median PFS was 7.1 months (95% CI: 4.2–9.9). Progression and mortality events were noted during follow-up. In univariable analyses, IDH1 expression exhibited an association with PFS; however, this association was not sustained after adjustment in the multivariable model. No variables showed a consistent association with PFS in multivariable analysis (Table 2). Effect estimates were imprecise, reflecting the limited sample size and event distribution. The membranous staining intensities (ranging from 0 to 3+) for CLDN18.2 in PDAC patients are illustrated in Figure 1. The evaluation primarily relied on basolateral and lateral membranous localization, deliberately excluding cytoplasmic reactivity from the scoring criteria.

Figure 2 presents representative immunohistochemical staining patterns for the IDH1 R132H mutation, utilizing the H09 clone. The staining indicates the presence of the mutant protein rather than the overall IDH1 expression, as evidenced by the granular cytoplasmic reactivity observed in the tumor samples depicted in panels C and D.

**Figure 3.**
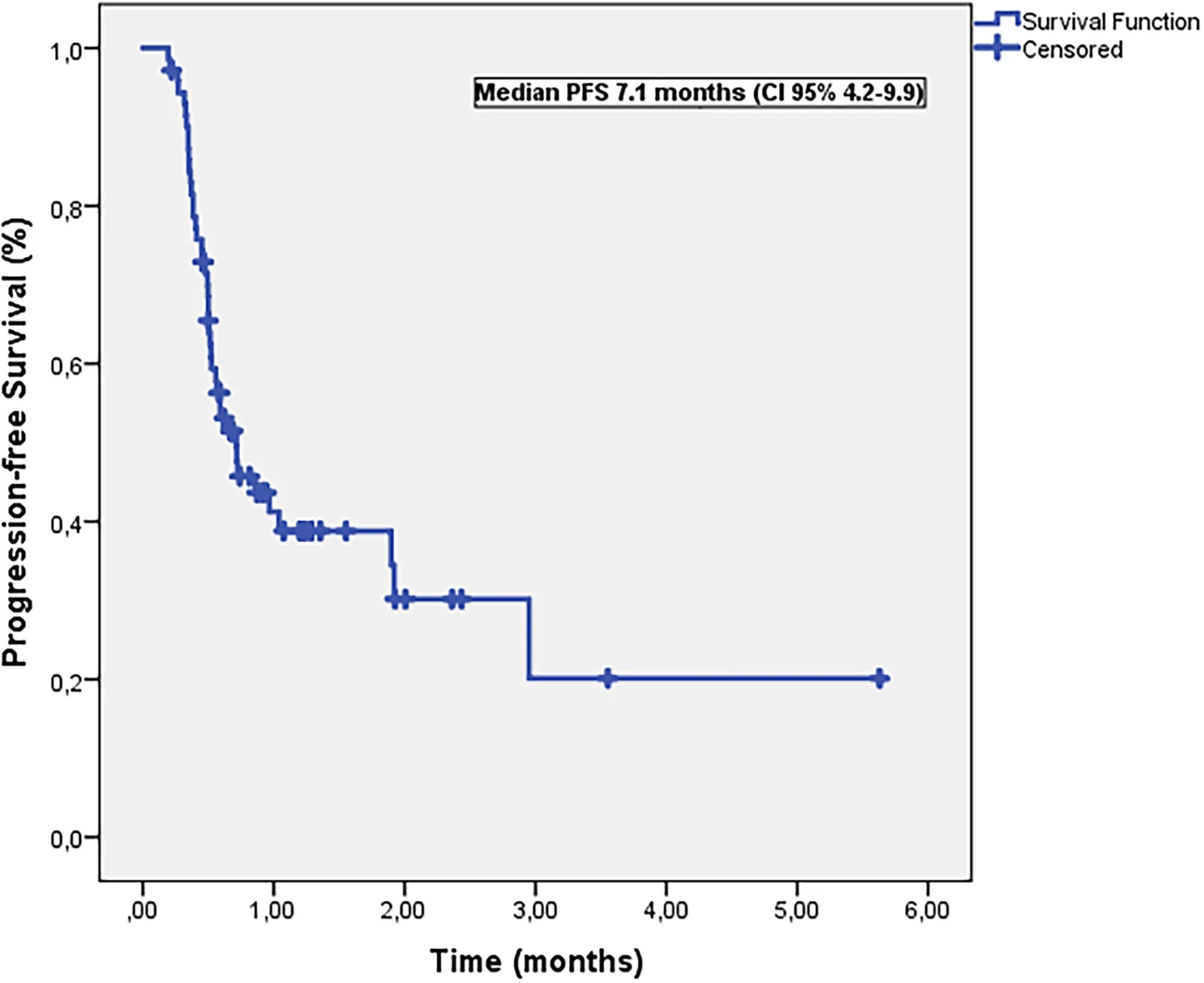
**Kaplan–Meier analysis of progression-free survival in the overall cohort.** The Kaplan–Meier curve shows PFS in 72 patients with unresectable PDAC receiving first-line chemotherapy. Median PFS was 7.1 months (95% CI, 4.2–9.9). Tick marks indicate censored observations. **Abbreviations:** CI, confidence interval; PDAC, pancreatic ductal adenocarcinoma; PFS, progression-free survival.

**Figure 4.**
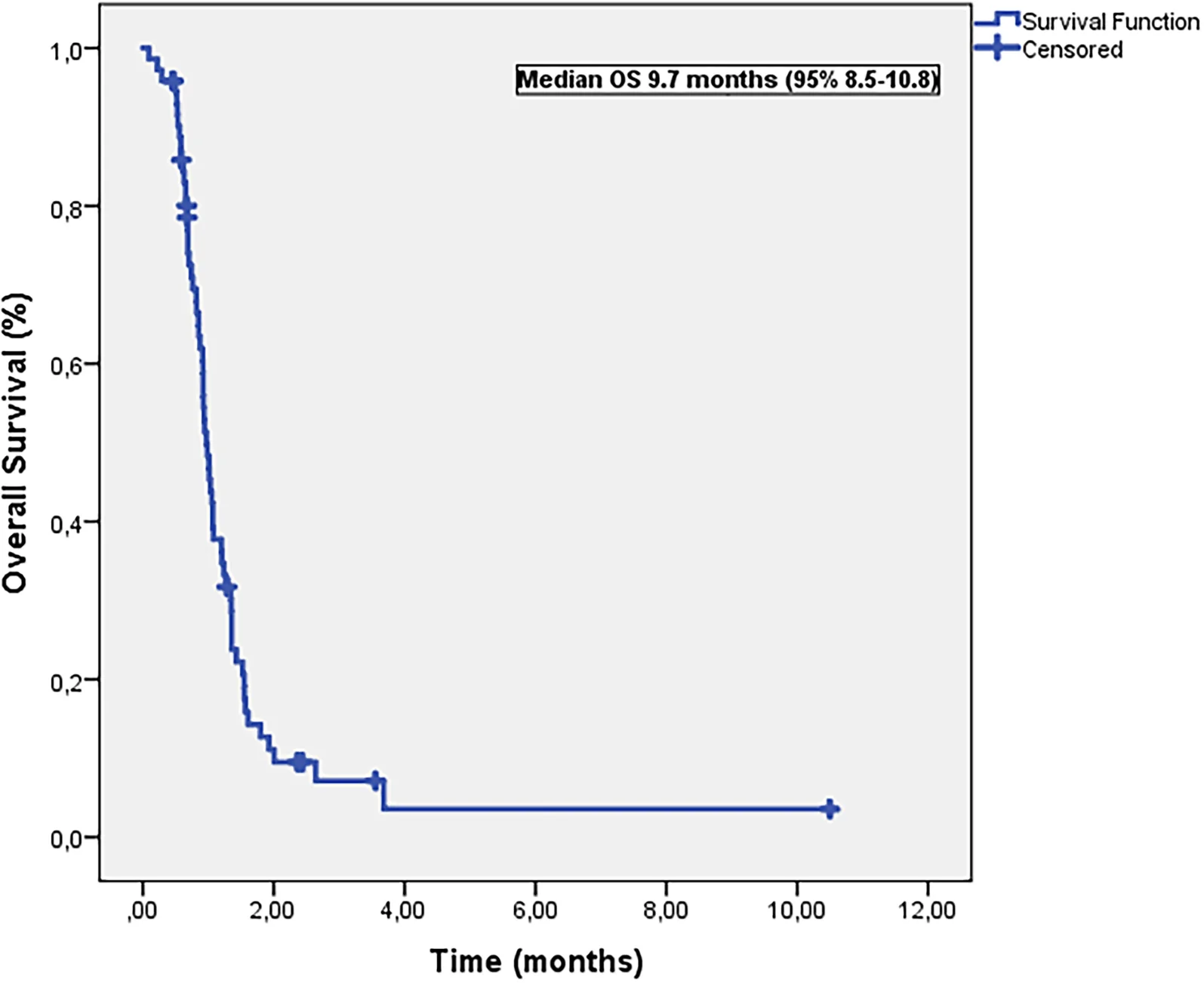
**Kaplan–Meier analysis of overall survival in the overall cohort.** The Kaplan–Meier curve shows OS in 72 patients with unresectable PDAC receiving first-line chemotherapy. Median OS was 9.7 months (95% CI, 8.5–10.8). Tick marks indicate censored observations. **Abbreviations:** CI, confidence interval; OS, overall survival; PDAC, pancreatic ductal adenocarcinoma.

### Overall survival

Kaplan–Meier analyses of the overall cohort are illustrated in Figures 3 and 4. The median follow-up duration, estimated using the reverse Kaplan–Meier method, was 5.7 months, indicating a relatively short follow-up period in this retrospective cohort. The median OS was 9.7 months (95% CI: 8.5–10.8 months; Figure 4). Death events occurred during follow-up, facilitating stable estimation of the Cox proportional hazards models. In univariable analysis, CLDN18.2 expression, IDH1 expression, and NLR were associated with OS. In multivariable analysis, these variables maintained their associations with OS (Table 3). CLDN18.2 positivity was linked to improved survival compared to the negative group. For IDH1, the effect indicated a lower hazard in the IDH1-positive group relative to the IDH1-negative reference category; however, this finding should be interpreted cautiously due to the very small number of IDH1-positive cases. NLR remained associated with OS in the primary analysis when modeled as a log-transformed continuous variable. This association was consistently observed in sensitivity analyses utilizing both the cohort median cutoff (3.22) and the clinically relevant cutoff (≥3).

**Table 3:** Multivariable Cox regression analysis for overall survival (full model output)

**Variable**	**Category**	**HR (95% CI)**	***P* value**
Age	>60 vs ≤60	1.21 (0.68–2.14)	0.51
Sex	Male vs Female	1.18 (0.66–2.10)	0.57
Smoking history	Positive vs Negative	1.12 (0.63–1.98)	0.70
Diabetes	Yes vs No	1.25 (0.71–2.21)	0.44
Metastasis at diagnosis	Yes vs No	1.36 (0.75–2.46)	0.31
Chemotherapy regimen	Gem+nab vs FOLFIRINOX	1.29 (0.72–2.31)	0.39
CLDN18.2 expression	Positive (reference)	–	–
	Negative	2.63 (1.23–5.55)	0.013
IDH1 expression	Negative (reference)	–	–
	Positive	0.68 (0.19–0.72)	0.039
NLR (continuous, log)	per unit increase	1.74 (1.12–2.71)	0.014
NLR (categorical)	≤3.22 (reference)	–	–
	>3.22	1.92 (1.03–3.56)	0.038

IDH1 results must be interpreted with caution as exploratory findings due to the limited number of positive events. Hazard ratios are reported in relation to the reference category. All variables included in the multivariable model are presented, irrespective of their statistical significance. **Abbreviations:** CI, confidence interval; CLDN18.2, claudin 18.2; FOLFIRINOX, folinic acid, fluorouracil, irinotecan, and oxaliplatin; Gem+nab, gemcitabine plus nanoparticle albumin-bound paclitaxel; HR, hazard ratio; IDH1, isocitrate dehydrogenase-1; NLR, neutrophil-to-lymphocyte ratio.

## Discussion

The primary findings of this study indicate that negative CLDN18.2 expression, positive IDH1 expression, and elevated NLR are linked to poorer overall survival in patients with unresectable PDAC undergoing first-line chemotherapy, whereas no significant associations were found for progression-free survival. These results suggest that combined molecular and systemic biomarkers may reflect underlying tumor-host interactions that influence long-term outcomes rather than short-term treatment responses. Given the retrospective design and limited sample size, these observations should be considered as hypothesis-generating.

Previous studies evaluating CLDN18.2 in PDAC have reported heterogeneous expression rates and inconsistent associations with survival [8–12, 23]. While certain studies demonstrated high expression rates [8, 9, 11], others noted lower prevalence and variable prognostic impact [10, 12]. These discrepancies may arise from differences in immunohistochemical methods, scoring systems, and patient populations. In our cohort, the relatively low CLDN18.2 positivity rate (18.1%) and its association with improved OS align with prior findings [12]. This may suggest that a lack of CLDN18.2 expression identifies a more aggressive tumor phenotype, although residual confounding related to disease stage cannot be excluded.

Although IDH1 expression was associated with OS, the direction and magnitude of this effect should be interpreted cautiously due to the very small number of IDH1-positive cases (*n* ═ 2).

We also observed that elevated NLR was associated with poorer OS. NLR showed consistency across continuous and categorical analyses, reinforcing its role as a marker of systemic inflammation. These findings are consistent with those of Kitsugi et al. [24], who demonstrated an association between elevated NLR and worse survival in advanced PDAC. However, NLR should be interpreted with caution, as it may be affected by non-oncological factors such as comorbidities or medications. Modeling NLR as a continuous variable provided more robust estimates, while categorical analyses were retained for clinical interpretability.

Regarding IDH1, its potential prognostic role may relate to its function in cellular redox regulation and in the production of reduced nicotinamide adenine dinucleotide phosphate (NADPH), which aids in resistance to oxidative stress [16, 25]. Targeting IDH1 represents a promising therapeutic strategy, with mutant IDH1 inhibitors such as ivosidenib currently under clinical evaluation [25, 26]. Concurrently, modulation of systemic inflammation reflected by NLR through anti-inflammatory approaches, neutrophil-targeted therapies, and immune checkpoint inhibition is an active area of investigation in PDAC [27–30]. Together, these findings suggest that biomarkers such as CLDN18.2, IDH1, and NLR may help identify biologically distinct patient subgroups, although their clinical utility requires further validation. Emerging data also indicate interactions between redox balance and ferroptosis-related pathways in PDAC progression [31]. However, due to the very low prevalence of IDH1 positivity in our cohort (2.8%), these findings should be interpreted cautiously, as the observed effect estimates may be unstable.

The lack of association between these biomarkers and PFS may reflect differences between short-term treatment response and long-term disease biology. While PFS is influenced by treatment efficacy and assessment variability [22], OS captures the broader interaction between tumor biology and host factors. This distinction may clarify why biomarkers reflecting systemic inflammation and tumor aggressiveness are more significantly associated with OS. The relatively short median follow-up duration may limit the interpretation of long-term survival outcomes.

This study has several limitations. Its retrospective, single-center design and limited sample size restrict generalizability. Immunohistochemical evaluation from single biopsy specimens may not capture tumor heterogeneity. The low number of IDH1-positive cases limits the robustness of this finding. Additionally, the absence of key clinical variables such as Eastern Cooperative Oncology Group (ECOG) performance status may have introduced residual confounding. Therefore, these results should be considered hypothesis-generating and require validation in larger, prospective studies.

Recent advances suggest that integrated inflammatory and computational biomarker models may enhance prognostic accuracy in cancer [32–34]. While our study focused on readily available markers such as NLR, future studies incorporating multi-dimensional approaches may further refine risk assessment in PDAC.

## Conclusion

Our findings indicate that CLDN18.2 and IDH1 expression, alongside NLR, may be associated with overall survival in patients with unresectable PDAC receiving first-line chemotherapy. These observations support a potential role for combined molecular and systemic biomarkers in prognostic assessment. However, given the retrospective design and limited sample size, the results should be regarded as hypothesis-generating and necessitate validation in larger, independent cohorts prior to clinical application.

## Data Availability

The data that support the findings of this study are available from the corresponding author upon reasonable request.
